# Outreach Training and Supportive Supervision for Quality Malaria Service Delivery: A Qualitative Evaluation in 11 Sub-Saharan African Countries

**DOI:** 10.4269/ajtmh.23-0316

**Published:** 2024-02-06

**Authors:** Robin Altaras, Matt Worges, Sabrina La Torre, Bala M. Audu, Grace Mwangi, Albert Zeh-Meka, Paul Yikpotey, Irenee Domkam Kammogne, Pascalina Chanda-Kapata, Caroline Vanderick, Joshua Yukich, Elizabeth Streat

**Affiliations:** ^1^ Tropical Health, Angers, France;; ^2^ Tropical Health, New Orleans, Louisiana;; ^3^ Tropical Health, Roquebilliere, France;; ^4^ Tropical Health, Abuja, Nigeria;; ^5^ Tropical Health, Nairobi, Kenya;; ^6^ Tropical Health, Yaoundé, Cameroon;; ^7^ Tropical Health, Accra, Ghana;; ^8^ Tropical Health, Niamey, Niger;; ^9^ Tropical Health, Lusaka, Zambia;; ^10^ Tropical Health, Barcelona, Spain;; ^11^ Tulane School of Public Health and Tropical Medicine, New Orleans, Louisiana;; ^12^ Tropical Health, Maputo, Mozambique

## Abstract

Quality improvement of malaria services aims to ensure that more patients receive accurate diagnosis, appropriate treatment, and referral. The Outreach Training and Supportive Supervision Plus (OTSS+) approach seeks to improve health facility readiness and provider competency through onsite supportive supervision, troubleshooting, and on-the-job training. As part of a multicomponent evaluation, qualitative research was conducted to understand the value of the OTSS+ approach for malaria quality improvement. Semistructured key informant interviews, focus group discussions, and structured health facility–based interviews were used to gather stakeholder perspectives at subnational, national, and global levels. Data were collected globally and in 11 countries implementing OTSS+; in-depth data collection was done in four: Cameroon, Ghana, Niger, and Zambia. Study sites and participants were selected purposively. Verbatim transcripts were analyzed thematically, following the Framework approach. A total of 262 participants were included in the analysis; 98 (37.4%) were supervisees, 99 (37.8%) were supervisors, and 65 (24.8%) were other stakeholders. The OTSS+ approach was perceived to improve provider knowledge and skills in malaria service delivery and to improve data and supply management indirectly. Improvements were attributed to a combination of factors. Participants valued the relevance, adaptation, and digitization of supervision checklists; the quality and amount of contact with problem-solving supervisors; and the joint identification of problems and solutions, and development of action plans. Opportunities for improvement were digitized checklist refinement, assurance of a sufficient pool of supervisors, prioritization of health facilities, action plan dissemination and follow-up, and data review and use. The OTSS+ approach was perceived to be a useful quality improvement approach for malaria services.

## INTRODUCTION

Quality improvement of malaria service delivery aims to ensure that more patients receive accurate diagnosis, appropriate treatment, and referral, as well as to improve the reporting of malaria cases and management of malaria supplies. Moreover, in high-burden countries, malaria is responsible for a large proportion of outpatient visits to health facilities and hospital admissions. In these contexts, quality improvement of malaria care and prevention services should, theoretically, help catalyze improvements in overall health-care quality.

Supportive supervision is one quality improvement intervention used to reach health-care providers where they practice, to enable them to offer quality services, and to improve their performance and motivation.^1^ Supportive supervision approaches are widely used, but are variously defined, implemented, supported, and sustained.^2^^–^^5^ Key elements of supportive supervision generally include open, two-way exchange and learning, provision of constructive feedback, and team-based or whole-site approaches that facilitate problem solving and are action oriented.^2^ Some countries use targeted supervision approaches, whereas others have shifted to integrated supportive supervision, which aims to improve the quality of primary health-care delivery across a range of diseases or health needs.^6^^–^^8^

Despite the wide use of supportive supervision approaches, evidence supporting their effect on improving service quality in different health areas has been limited, with only modest or no gains observed in some contexts.^1^^,^^9^^–^^12^ Low-quality evidence has been a reported challenge^12^; another possible explanation for limited evidence of effectiveness has been a failure to address systemic requirements, such as sufficient numbers of skilled human resources and supply chain management.^10^ In addition, challenges with designing, implementing, and sustaining supportive supervision approaches may limit long-term effects. An absence of feedback after visits, irregular visits, poor continuity between visits, top-down planning, and a failure to adhere to supervision plans because of inadequate resources for implementation have all been reported challenges.^13^^,^^14^ Historically, unsupportive or authoritarian supervisory approaches have also been perceived to generate negative effects, with some evidence that negative feedback or unsupportive supervision may be more demotivating than no supervision at all.^13^^,^^15^

The Outreach Training and Supportive Supervision (OTSS) approach is a facility-level approach aimed at improving health facility readiness and provider competency through onsite supportive supervision, troubleshooting, and on-the-job training. First developed in 2007 under the U.S. President’s Malaria Initiative (PMI) Improving Malaria Diagnostics Project, the approach was continued under the MalariaCare Project from 2012 to 2017. Beginning in 2018, the Impact Malaria (IM) Project rolled out an enhanced version of the approach—Outreach Training and Supportive Supervision Plus (OTSS+)—that introduced improvements based on a review of OTSS operational considerations. The history and scope of the approach are described in the companion paper by Barat et al.^16^

The OTSS+ approach aims to achieve an appropriate and measurable level of health worker competence in the management of uncomplicated and severe malaria, malaria in pregnancy, and performance of malaria rapid diagnostic tests (RDTs) and malaria microscopy. The approach also measures health facility readiness to verify that health facilities have the necessary supplies, infrastructure, and human resources to provide quality malaria services.

With the OTSS+ approach, trained supervisors observe providers, review records, and assess health facility readiness using a set of seven standardized, competency-based, and digitized checklists. Countries select and adapt the OTSS+ checklists in line with their specific policy and system needs. Not all countries use all checklists. Some countries have adapted OTSS+ checklists as part of an integrated supportive supervision checklist (Ghana), whereas others integrated OTSS+ into an existing mentoring checklist (Kenya). Provider performance is scored on several key steps in the clinical management of febrile illness. There is a minimum acceptable score (usually 90%) for a provider to be deemed competent in a specific area. Supervisors provide same-day, on-the-job training and feedback. Identified issues are discussed in a debrief session with the supervisees, health facility in-charges, and supervisors, who formulate an action plan jointly to address problem areas. Additional mentoring, coaching, or training may then be planned. The definition and use of mentoring and coaching approaches vary across countries, with some overlap in the use and meaning of terms. Mentoring typically involves the development of a long-term, sustained relationship to support capacity development. The OTSS+ approach rounds of visits are typically planned either quarterly or biannually. In between OTSS+ rounds, data review meetings and lessons-learned workshops are held at the regional, district, or subdistrict level to review OTSS+ visit data, action plans and follow-up, and malaria indicators; and to prioritize health facilities for the next round of visits.

During the MalariaCare Project, the OTSS approach was found to improve health worker performance on clinical case management of febrile patients, RDT performance, and malaria microscopy after three OTSS visits.^17^^–^^19^ Although clinicians showed good baseline knowledge on the clinical management of malaria, the OTSS approach was able to identify areas needing further improvement, such as health worker behaviors associated with assessing the severity of illness, managing nonmalarial fevers, and communicating effectively between patient and provider.^17^ A small number of evaluations of supervisors found that overall performance was high, but that development and follow-up on action plans was inconsistent.^20^ In Zambia, health facilities receiving OTSS visits showed improvements with regard to diagnostic skills, laboratory best practices, fever case management practices, and prescriber adherence to negative malaria test results.^21^

There has been little qualitative evidence regarding perceived benefits derived from the OTSS+ approach and which components of the approach contribute to or limit success. This study elicited the perspectives of supervisors, supervisees, managers, and other malaria and health system stakeholders on the value of the OTSS+ approach for improving the quality of malaria service delivery. In addition, the study sought to identify opportunities for strengthening the approach from the perspective of key implementers.

## MATERIALS AND METHODS

### Study design.

This qualitative study was conducted as part of a multicomponent, independent evaluation of the effectiveness of the OTSS+ approach, which involved quantitative OTSS+ and health management information system data analysis, qualitative research methods, and a cross-sectional online survey of OTSS+ supervisors and other stakeholders. This article presents findings from the qualitative research. The quantitative component of the evaluation is reported separately.^22^

To design the study, we first developed a retrospective theory of change for the OTSS+ approach. The theory of change acknowledged numerous contextual factors that may influence health worker decision making and malaria case management to varying degrees: patient profiles and demands, patient care-seeking and risk perception, difficult access to care for follow-up, high malaria prevalence in the patient population, lack of a reliable supply of tests and drugs (rationing of testing), health system functionality and resources, and constraints related to geography, insecurity, environment, natural disasters, and pandemics. We theorized that for the approach to be effective as designed, the following would need to be true in each context: the country and health system were well engaged in the adoption and adaptation process, the provided support was appropriate and responsive to health worker needs, supervisors had the necessary qualities and capacities to be able to catalyze improvements, the right health facilities and health workers were targeted, the right amount of support was provided for both efficiency and effectiveness, and the right data were available and acted on (Table 1). These theory-based domains were used to develop domains of inquiry and were included in an overall evaluation matrix that encompassed broader domains such as relevance, effectiveness, efficiency, and sustainability. Qualitative research methods were semistructured key informant interviews (KIIs) at global, national, and subnational levels; focus group discussions (FGDs) at the subnational level; and structured health facility–based interviews focused on reviewing experience and outputs from the most recent OTSS+ visit.

**Table 1:** Theory of change assumptions

Assumption	Example
The country and health system are well engaged in the adoption and adaptation process.	Health facilities and districts are involved, feel ownership for, and help drive the quality improvement process. Checklists capture the right information, balancing time/workload and content. Tools are linked to wider quality improvement processes and feedback loops at all levels, including citizen/patient feedback and health worker feedback. Tools are integrated across health areas as appropriate.
The provided support is appropriate and responsive to health worker and health facility needs.	Appropriate adult learning and mentoring methods are used for health worker behavior change. Health worker and health facility needs are addressed. The provided support “helps them get their job done.” Contextual factors and drivers of health worker decision-making and prescribing behavior are understood and addressed. Supervision and support avoid disruption of health facility work.
Supervisors have the qualities and capacity to catalyze improvements.	A sufficient pool of qualified cadres is available. Supervision team composition and numbers provide optimal benefits (effectiveness and efficiency) per health facility and health worker. There is effective interplay between laboratory and clinical supervisor cadres. Supervisors have the necessary mentoring and relationship-building skills. Onboarding of newly trained supervisors occurs as needed. Supervisors are comfortable using digital tools for analysis and action. Attention is given to equity and gender equality. There is timely disbursement of funds for visits.
The right health facilities and health workers are targeted.	Health facilities and health workers are targeted for the greatest benefit. Resources are allocated optimally based on health facility performance, level, and staffing. Health worker attrition and rotations are addressed; new staff are onboarded. Attention is given to equity and gender equality.
The right amount of support is provided for both efficiency and effectiveness.	Visit length is sufficient for both data collection and onsite training/support. Duration and frequency of visits and support is appropriate to improve and maintain performance. The point of diminishing returns is identified to support efficient resource allocation. Supervision visits and mentoring resources are prioritized for the most benefit. Environmental and external factors allow sufficient visits and interaction.
The right data are available and acted on.	Electronic tools match program needs, are easy to use, and require minimal support. Event- and case-based data are collected, analyzed, and used to improve the course of action and to allow prioritization of resources. Feedback is shared and acted on regularly at all levels of the health system.

### Study setting.

**Figure 1.**
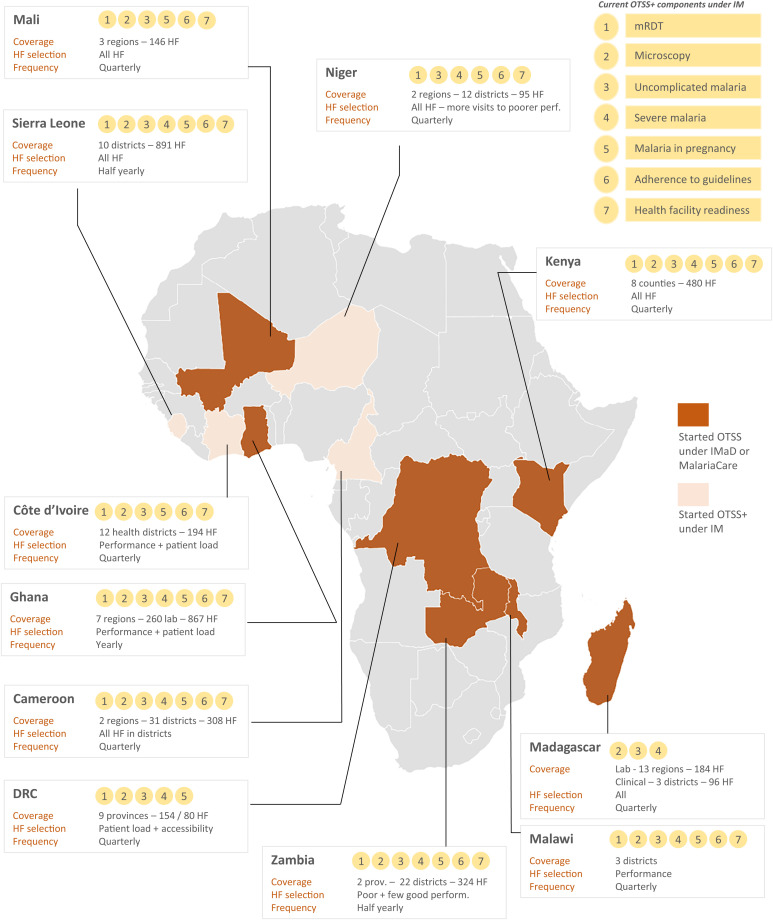
Overview of Outreach Training and Supportive Supervision Plus (OTSS+) history, coverage, and approach in each country. HF = health facilities; IM = Impact Malaria Project; IMaD = Improving Malaria Diagnostics Project; lab = laboratories; mRDT = malaria rapid diagnostic test.

The evaluation was conducted in 11 countries in sub-Saharan Africa, 10 of which were currently implementing the OTSS+ approach under the IM Project and one (Cote D’Ivoire) in which the IM Project closed in 2021. Although the OTSS+ approach uses a standardized framework, implementation of the approach varies across countries. The services covered, type of follow-up used, geographic scope, prioritization of health facilities, and frequency of OTSS+ rounds all may be adjusted on a country basis (Figure 1). Four countries (Cameroon, Ghana, Niger, and Zambia) were selected purposively for in-depth data collection in line with the principles of maximum variation sampling to identify countries that differed with regard to the scope of OTSS+ implementation (services covered), length of implementation experience (historical involvement versus more recent adoption), and linguistic/geographic region. Other factors that were considered in consultation with stakeholders were willingness and availability to support implementation of the evaluation, use of the standardized OTSS+ approach and limited use of complementary approaches, and the completion of at least three rounds of OTSS+ visits at the time of study. The remaining seven countries were included in the study via a limited number of KIIs with national- or regional-level stakeholders. In addition, the study was conducted at a global level, targeting stakeholders located in Africa, North America, and Europe.

### Selection of study sites.

In the four selected countries, district and health facility study sites were selected purposively, as mentioned earlier, for in-person data collection using a three-stage process. One region or province was selected from all areas implementing the OTSS+ approach in the past 6 months based on feasibility, access, and health and security concerns. Within the selected region or province, all districts participating in the last round of the OTSS+ approach were identified; preference was then given to districts that had completed multiple rounds of the OTSS+ approach during the IM Project. Among eligible districts, two or three districts were selected purposively for a range of OTSS+ supervision teams (districts covered by different supervision teams) and for operational feasibility (easily accessible within a predetermined field time of 10 days and no security concerns). At least one of the districts had to include a referral facility offering severe malaria case management services.

Within selected districts, four to six health facilities were selected purposively to achieve a range of OTSS+ performance trajectories. First, all health facilities receiving multiple OTSS+ visits were identified. Using the scores from the OTSS+ visit data from the last two rounds, all health facilities were then assigned one of three performance trajectories (stable, worsening, or improving) based on the difference between performance during the past two visits. (We used the last two rounds because, in some countries, few health facilities had received more than two visits.) “Stable” was defined as a difference of less than 10% in either a positive or negative direction; “worsening,” as a difference of 10% or more in a negative direction; and “improving,” as a difference of 10% or more in a positive direction. At least two health facilities were then selected randomly from each performance category. All samples were confirmed to include a range of health facility types and services provided, and to be less than a 2-hour drive to a central location. Three health facilities in one district (one hospital and two clinics) were targeted for health facility–based interviews focused on reviewing experience and outputs from the most recent OTSS+ visit; these were selected based on midrange proximity (not clustered around the district headquarters) and ease of research team access.

In addition, in Cameroon, Niger, and Zambia, the second region implementing the OTSS+ approach in the past 6 months was targeted for limited remote KIIs; in Ghana, an additional three regions were targeted for remote KIIs.

### Selection of study participants.

The study population included stakeholders involved in OTSS+ planning, implementation, and funding at subnational, national, and global levels; subnational was divided into regional, district, and health facility levels. Prospective stakeholder lists were drawn up in consultation with the IM Project and PMI country teams.

Study participants at global, national, and regional levels were selected purposively for KIIs based on a high level of involvement in the OTSS+ approach, including, where relevant, historical involvement, and to achieve representation of the different OTSS+ planning and implementation roles. Within each of the field study sites, district and health facility actors were selected purposively for KIIs or FGDs based on participation in an OTSS+ visit in the past 6 months, cadre, and gender balance, when feasible. In health facilities selected for the health facility–based KII and OTSS+ visit review, the health facility in-charge and one to two health workers who participated in the supervision visits or the implementation of subsequent quality improvement actions were targeted for a KII and review of action plans.

An estimated 25 KIIs were planned per country in the four in-depth countries and up to four KIIs in each of the remaining seven countries. An estimated six to eight FGDs involving six to eight participants were planned in the four countries. Focus group discussions were segmented by OTSS+ and health system roles: 1) OTSS+ district and regional supervisors, 2) district or regional health management team members, and 3) supervisees (health workers) by service delivery role (clinicians, laboratory staff, and antenatal care [ANC] staff). In Ghana, an additional eight KII participants from two additional regions were targeted, in line with a request from the national malaria control program.

### Data collection instruments.

Six data collection instruments were developed for the different respondent groups: two semistructured interview guides, three FGD topic guides, and one structured interview questionnaire for health facility–based KIIs (Table 2). The latter included a request to review the outputs and follow-up actions from the most recent OTSS+ visit. English versions of the tools were translated to French. English versions were piloted with national-level OTSS+ stakeholders not sampled for data collection in Zambia and Ghana, and French versions were piloted in Cameroon; key terms and concepts were reviewed and revised subsequently.

**Table 2:** Data collection methods and domains of inquiry

Data collection instrument	Domains
Semistructured interview guide for national- and subnational-level (regional or district) participants*	Checklist adoption and adaptation
	Digital tools and data use
	Effect on the quality of malaria service delivery
	Sustainability and institutionalization
	Learning topics: scope of supervision visits and specific approaches for improving health worker and health facility performance, supervisor capacities and team composition, health facility visit prioritization and frequency
	Evolution of the approach over time and lessons applied to newer countries (global participants)
FGD topic guides for supervisors, supervisees, and district health managers	Impact of OTSS+ on job performance (supervisees)
	Impact of OTSS+ on quality of care at health facility
	Aspects of OTSS+ providing the most benefit
	Aspects of OTSS+ that are not helpful or unnecessary
	Relevance and value of OTSS+ for addressing health facility needs
	Action plan development and follow-up
	Feedback provided during or after OTSS+ visit
	Specific strategies used to support health facilities and health workers
	Supervisor qualities and capacities
	Supervisor experience, training, and performance
	Use of digital tools and data (supervisors, managers)
	Health facility selection and prioritization (managers)
	Areas for improvement of the approach
Structured interview questionnaire for health facility in-charges or other staff	OTSS+ implementation experience
	Perceived outcomes and value of the OTSS+ visit
	Use of digital tools
	Institutionalization of OTSS+ and quality improvement processes within the health structure
	Review of outputs and follow-up actions from the most recent OTSS+ visit

FGD = focus group discussion; OTSS+ = Outreach Training and Supportive Supervision Plus.

* The core instrument was modified for global participants.

### Data collection procedures.

Data collection was carried out from April to June 2022 over an approximately 10-day period in each country. Data were collected by 11 research team members experienced in qualitative data collection: two-person teams conducted KIIs and FGDs in the four countries (led by A. Z., P. C., P. Y., and I. K.), and three people (E. S., S. L., and B. A.) conducted interviews globally and in the remaining seven countries. All data collection team members participated in 1 day of remote orientation and training sessions covering ethics, data collection tools, and core topic areas; transcription; and COVID-19 prevention protocols.

Selected participants were invited for interview or FGD participation by district health officials or via phone outreach from the research team. Interviews and FGDs were 45 to 60 minutes long, conducted in English or French, and were audio recorded. All FGDs were held in person; participants from the selected study sites were requested to travel to a central location in the district. Key informant interviews were conducted either in person (*n =* 37) or virtually (*n =* 96), depending on participant location, availability, and health or security requirements. Semistructured interview topics were prioritized in line with the participant’s area of expertise, role, and involvement in the OTSS+ approach, and, as data collection progressed, areas requiring further exploration. Data collection continued until theoretical saturation was reached in each of the four countries. In total, 133 KIIs and 22 FGDs were conducted (Table 3).

**Table 3:** Data collection

Country	KIIs completed, *n*	KII participants, *n*	FGDs completed, *n*	FGD participants, *n*	Total participants, *n*	Participants retained for analysis, *n*
Cameroon	21	21	5	25	46	46
Ghana	32	32	6	42	74	74
Niger	23	24*	5	26	50	50
Zambia	21	21	6	38	59	51
Cote d’Ivoire	4	4	N/A	N/A	4	4
Democratic Republic of the Congo	2	2	N/A	N/A	2	2
Kenya	4	4	N/A	N/A	4	4
Madagascar	3	3	N/A	N/A	3	3
Malawi	4	4	N/A	N/A	4	4
Mali	3	3	N/A	N/A	3	3
Sierra Leone	3	3	N/A	N/A	3	3
Global	13	18*	N/A	N/A	18	18
Total	133	139	22	131	270	262

FGD = focus group discussion; KII = key informant interview; N/A = not applicable.

* Three KIIs were conducted with multiple participants; two KIIs (in Niger and global) had two participants, and one global KII had five participants.

### Data management and analysis.

Audio recordings of all KIIs and FGDs were transcribed using voice-to-text software: Otter.ai (AISense, Inc., Mountain View, CA) for the English language and Sonix (Sonix Inc., San Francisco, CA) for French. Transcripts were quality-assured by A. Z., P. K., P. Y., and I. K. Three transcripts from Zambia were excluded from analysis because of poor-quality transcription—two KIIs (two participants) and one FGD (six participants)—resulting in a final sample of 152 transcripts and 262 participants (Table 3). French transcripts were translated into English using DeepL or Doc Translator, and translations were reviewed and edited for accuracy by a bilingual research assistant briefed on the scope of data collection and country contexts. All transcripts were first read in full by a senior researcher (E. S.) and then uploaded into MAXQDA 2022 (version 22.2.0) for coding and thematic analysis. Coding was conducted primarily by four researchers (E. S., M. W., S. L., and G. M.), who met regularly to review and discuss coding decisions with R. A. Thirteen transcripts (8.6%) were dual-coded, with discrepancies identified and discussed to reach consensus.

Thematic analysis followed the Framework approach.^23^ An initial coding frame was developed based on the scope of inquiry and to which codes were added as review of data progressed. Coded KII and FGD transcripts were pooled for thematic analysis, which was done for efficiency and to facilitate an analytical focus on participant categories—both health system and OTSS+ roles. Subthemes were then summarized by country and by participants’ OTSS+ roles, with particular attention to triangulating supervisor and supervisee perspectives. Summary functions within MAXQDA were used to group data by categories and subcategories to facilitate the process of analysis and interpretation. In this article, we focus on presenting a thematic summary across countries, with specific attention paid to the perspectives of participants involved most directly in OTSS+ implementation.

## RESULTS

### Study participant characteristics.

Of 262 study participants, 108 (41.2%) were women, 98 (37.4%) were supervisees, 99 (37.8%) were supervisors, and 9 (3.4%) were district or regional-level managers. The remainder constituted a range of national and other stakeholders (Table 4).

**Table 4:** Characteristics of study participants, by gender, included in the analysis

Characteristic	Male		Female		Total	
	*n*	%	*n*	%	*n*	%
Total participants	154	58.8	108	41.2	262	100
Health system level						
Health facility	38	40.9	55	59.1	93	35.5
District	48	71.6	19	28.4	67	25.6
Regional	37	80.4	9	19.6	46	17.6
National	22	57.9	16	42.1	38	14.5
Global	9	50.0	9	50.0	18	6.9
Country OTSS+ role						
Supervisee	41	41.8	57	58.2	98	37.4
Clinical staff	26	47.3	29	52.7	55	56.1
ANC staff	4	16.7	20	83.3	24	24.5
Laboratory staff	11	57.9	8	42.1	19	19.4
Supervisor	72	72.7	27	27.3	99	37.8
Supervisor/clinician	43	69.4	19	30.6	62	62.6
Supervisor/trainer	2	100.0	0	0.0	2	2.0
Supervisor/manager	14	70.0	6	30.0	20	20.2
Supervisor/data manager	13	86.7	2	13.3	15	15.2
Manager	8	88.9	1	11.1	9	3.4
National government stakeholder*	11	61.1	7	38.9	18	6.9
Implementing partner	14	60.9	9	39.1	23	8.8
Other stakeholder^†^	8	53.3	7	46.7	15	5.7
Country						
In-depth countries						
Cameroon	33	71.7	13	28.3	46	17.6
Ghana	50	67.6	24	32.4	74	28.2
Niger	24	48.0	26	52.0	50	19.1
Zambia	24	47.1	27	52.9	51	19.5
Remaining countries						
Cote D’Ivoire	2	50.0	2	50.0	4	1.5
Democratic Republic of the Congo	1	50.0	1	50.0	2	0.8
Kenya	1	25.0	3	75.0	4	1.5
Madagascar	1	33.3	2	66.7	3	1.1
Malawi	4	100.0	0	0.0	4	1.5
Mali	3	100.0	0	0.0	3	1.1
Sierra Leone	2	66.7	1	33.3	3	1.1
Global	9	50.0	9	50.0	18	6.9

ANC = antenatal care; OTSS+ = Outreach Training and Supportive Supervision Plus.

* National government stakeholder refers to all central-level government stakeholders, including mentors and trainers.

^†^ Other stakeholder refers to all other partners, including funders.

### Perceived value of the OTSS+ approach for quality improvement in malaria case management and prevention.

Stakeholders at all levels perceived that the OTSS+ approach contributed to improving health facility readiness and health worker performance in delivering malaria services. Enhanced provider awareness, knowledge, and skills was perceived to improve providers’ performance with regard to differentiating severe and uncomplicated malaria, correct use of diagnostic tools for confirmatory diagnosis, adherence to national guidelines, and interaction with patients. In addition, the OTSS+ approach was perceived to have an indirect impact on the health system, leading to improved documentation and supply management. These themes are described next.

#### Provider awareness, knowledge, skills, and confidence.

The OTSS+ approach was perceived to lead to increased awareness, more in-depth knowledge, and increased confidence in the performance of both clinical and laboratory staff. Some providers described developing a deeper understanding of the theoretical reasons behind different clinical and diagnostic (microscopy and RDT) procedures, noting that supervisors took the time to explain. They in turn perceived that this understanding made them more inclined to follow standard procedures and guidelines, such as checking the RDT expiration date, wearing gloves, and waiting the right amount of time before declaring a test negative. A regional supervisor in Ghana noted that the system compels them to learn how to do “a lot of things they are not doing.” Focus group participants from Cameroon and Ghana also cited improvements in understanding the need to carry out a thorough clinical assessment before testing. Similarly, supervisors and ANC staff described improved adherence to malaria-in-pregnancy chemoprophylaxis protocols, such as directly observed therapy and increased awareness of malaria-in-pregnancy treatment guidelines, including correct antimalarial dosing and dose preparation.

*[P]eople had a very poor grasp of [microscopy] techniques. There were many cases of malaria that were not malaria. But today, I can really reassure you, that about 95% of the cases tested positive are really malaria. So, it really changed a lot—everyone’s mentality.’ (Male, Laboratory Technician and Regional Laboratory Supervisor, Cameroon [KII 092])*

Supervisors and supervisees also reported improved awareness and adoption of test-before-treat, and an improved awareness of the importance of differential diagnosis, particularly as a method to diagnose appropriately and forego the use of artemisinin-based combination therapies in patients who test negative. In addition, supervisors and supervisees perceived improvements in the differentiation of uncomplicated and severe malaria based on clinical assessment; in particular, participants in Cameroon mentioned noticeable reductions in the proportion of cases diagnosed as severe following OTSS+ visits. Participants also mentioned an increased appreciation of the importance of reserving drugs and formulations intended for the treatment of severe cases, and not using them to treat uncomplicated malaria.

*[W]e couldn’t tell the difference between severe and simple malaria. And when we had cases of simple malaria, we gave the treatment of severe malaria. During the supervision, he showed how we must differentiate. (Female, Facility MCH Nurse Aide and Supervisee Cameroon [KII 081])*

*[I]f you’re qualifying malaria to be complicated, it means that you also must [record] what complication there [is] . . . [and] it has opened the eyes of the clinicians . . . to search for more . . . [and] that’s a very, very important impact on us. (Female, Health Facility In-Charge and District Supervisor, Zambia [KII 066])*

Supervisors and health facility in-charges reported increased confidence in health worker performance. In some cases, increased knowledge and procedural improvements were also perceived to lead to increased confidence levels of clinicians and laboratory personnel to conduct their work competently. There was also some mention of increased provider confidence in the performance of their colleagues. In Ghana, an OTSS+ supervisor noted that, following OTSS+ visits, clinicians were more inclined to send patients to the laboratory for a confirmatory diagnosis.

#### Patient–provider interaction.

Providers commented that OTSS+ guided them to improve patient reception, patient flow, and patient-provider communication during the consultation. They perceived that these changes had in turn led to increased trust between the patient and provider, and greater patient satisfaction, which some providers observed translated to increased attendance or return use.

*If the patient comes, you give him the RDT, you welcome him, and tomorrow he will go and say [to others in the community], “Go. There is [good] reception [at the health facility].” He’s happy. You are happy too. (Female, Nurse and Supervisee, Niger [KII 019])*

*Precisely, because the first thing is the reception of patients. I think that long before we already insisted a lot on the reception, because a well-received patient already feels safe. (Male, Health Facility In-Charge and Supervisee, Cameroon [KII 082])*

#### Provider motivation.

Providers noted their own increased satisfaction and motivation from providing improved quality of care and from observing positive health outcomes and satisfied patients. Increased provider motivation was also tied to the positive and encouraging nature of the supervisor–supervisee interaction, a key factor valued by participants.

*[Y]ou used to see malaria-in-pregnancy going high . . . and sometimes you see miscarriages because of complicated malaria. But with [OTSS+], it has reduced, and it motivates me because there’s no need to be running to save two lives because of malaria that I could handle by using maybe the ‘net, the SP [sulfadoxine-pyrimethamine], and the drug with my little education. So I think as [an] individual I would say [OTSS+ is] motivating. (Female, Midwife and Supervisee, Ghana [KII 030])*

#### Inventory management and data registries.

Supervisees, supervisors, and managers across the four countries noted two areas in which the OTSS+ approach had an indirect positive effect: inventory management and data registries. The latter was also perceived to have led to improved timeliness and completeness of data submission to the districts or health areas. These improvements were noted predominantly in the ANC unit and laboratory services, including RDT use. Managers also observed improvements in the quantification of supplies.

*There has been a particularly good interaction between the central staff or a provincial officer on logistics for Coartem and RDTs for testing malaria. (Female, Provincial Nursing Officer and Supervisor, Zambia [FGD 16-02])*

*With the supervision, all the ANC data are currently [recorded]. It has helped us a lot. Even the new ones who entered the service recently, they can already fill in the register, because the last supervision has helped us a lot. (Male, Nurse Aide and Supervisee, Cameroon [FGD 20-01])*

### Factors valued by participants.

#### “The whole process.”

Participants viewed the OTSS+ approach as a package of different components, implemented in a systematic and sustained way. When asked which aspects of the approach provided the most benefit, a common theme was that the approach was a holistic process of improvement, and that “the whole package” drove effectiveness. Within this process, quality improvements were attributed to a combination of factors. We grouped these factors into three broad thematic areas: checklist relevance, adoption, and digitization; the quality and amount of supervisory contact; and joint problem identification, solving, and action planning (Table 5).

**Table 5:** Main themes, factors valued by participants, and perceived opportunities for improvement

Thematic area	Factors valued	Opportunities for improvement
Appropriate checklists/tools	Relevant checklists adapted for context: encouraging good country engagement and ownership of the adoption and adaptation process	Optimizing checklists after digitization: Inflexibility of binary data entry, skip patterns, and required fields affect provider scoring.
	Checklist digitization: sharing real-time results was perceived to enhance objectivity and transparency	
Quality and amount of supervisory contact	Onsite support: providing same-day coaching, mentoring, and other support	
	Helpful supervisors: using qualified, skilled, and supportive supervisors focused on problem solving	Ensuring a sufficient pool of supervisors: An insufficient number of supervisors was noted in some countries. Some perceive that more district-level supervisors should be trained.
	Length and maintenance of supervisory contact: having adequate time at the health facility during OTSS+ visits; planning and making follow-up contacts or visits	Prioritizing health facilities: Health facility selection was influenced by context, health system, and resource constraints. Given limited resources, it is unclear whether to prioritize follow-up visits or to reach new health facilities.
		Determining how much support is enough: The optimal number and frequency of supervision visits to achieve adequate competency is unclear. Context-specific decision making regarding the use of different types of follow-up (e.g., integrated, remote, same supervisor or not) is needed.
Joint identification of problems and solutions	A collaborative approach: creating an overall environment of openness, mutual learning, and trust	
	Action planning process: developing and agreeing jointly to a feasible plan based on local context	Action plan dissemination and follow-up: There is poor availability of action plans at health facilities. Digitized action plans lack specificity. Unclear follow-up plans were noted. There is a need for monitoring action plan implementation.
	–	OTSS+ data review and use: Actors could increase the use of OTSS+ data for planning malaria interventions outside of OTSS+ visits, particularly at the lower levels.

OTSS+ = Outreach Training and Supportive Supervision Plus.

#### Relevant checklists adapted for context.

Most supervisors, managers, and government stakeholders felt the OTSS+ checklists provided a relevant and adequate framework for quality-of-care improvement, and that the OTSS+ approach had been well adapted at the country level. They described multiphase processes of engagement with national malaria control programs and good country ownership of the process for reviewing and adapting the checklists and digital tools. As a result of these processes, national stakeholders across countries felt they had the flexibility to adapt the standardized tools as needed for their context. Examples of context-specific changes included dropping or modifying questions that were not applicable given national malaria case management guidelines, adapting to changes in guidelines over time, modifying questions that were linked to ensure accurate identification of gaps (for example, health worker behavior and the availability of required supplies such as gloves), and reformulating questions to align with local health facility realities and customs (for example, with regard to patient reception and health worker introduction). Strong country ownership and engagement were in turn perceived to support sustainability and institutionalization of the approach. Under the IM Project, newer countries were perceived to have benefited from intercountry sharing of experiences and a stepwise approach to accelerate implementation and adoption.

*So you went through the checklist and made some changes, tweaks . . . . It wasn’t significant tweaks, but it was small changes adapted to the local context, national contexts, and regional context. So we had meetings and then workshops with that. (Female, Other Stakeholder and Technical Advisor, Cameroon [KII 093])*

Checklist digitization was also perceived to enhance objectivity and transparency, because checklist data could not be manipulated and could be shown directly to the health facility staff.

*[W]ith the paper-based system, too, it made assessment more subjective than objective, but with this [digital tool], in all facilities it is the same criteria, because it’s programmed. If they are performing, it will show it [on the screen]. [If] they are not performing . . . , it will come and you’ll be able to give prompt feedback. And the feedback to me is very objective. (Male, Health Information Officer and Regional Supervisor, Ghana [KII 045])*

#### Quality and amount of supervisory contact.

Participants valued the onsite provision of coaching, mentoring, feedback, and other support; the use of qualified and skilled supervisors, who focused on collaborative problem-solving based on the local context; and the length and maintenance of supervisory contact.

##### Onsite, same-day support.

A major theme was that OTSS+ supervisors “don’t wait to solve problems,” but provide onsite observation and same-day support using a variety of different methods for knowledge sharing, depending on the context. The use of multiple and reinforcing channels was seen as differentiating the approach from other supervision schemes. Coaching and training onsite were also mentioned frequently as a means for ensuring that guidance reached more health workers. Supervisors also valued digitization of the checklists, which was perceived to simplify the process of observation and immediate feedback, because the electronic tools provided real-time scores and facilitated on-the-spot feedback.

*By bringing people to a central location to train, you have a lot of people around, some even come and sit, and they don’t listen to anything . . . . Getting the people to understand using the things they have onsite to teach them is the better approach. (Male, Health Information Officer and Regional Supervisor, Ghana [KII 033])*

##### Helpful, problem-solving supervisors.

Supervisors and supervisees felt that “good OTSS+ supervisors” embodied both technical competencies and strong interpersonal communication skills, which were underpinned by specific attitudes. Technical capacities were having “superior” knowledge, skills, and experience in malaria control; being able to “solve complicated problems on the spot,” answer questions, and give the right advice; understanding the context in which people work and not just knowing the “theory”; and having appropriate clinical qualifications and responsibilities.

*We don’t go into the field to look for problems, it’s to solve, to support the service provider . . . . We have to solve [problems] on the spot because that’s also our job. You don’t go down to collect [and] come back with problems to solve; no, you have to solve the problems. (Male, Nurse and Regional Supervisor, Cameroon [KII 091])*

Interpersonal communication skills included being a good listener and observer, and knowing how to probe to obtain “the real picture”; setting supervisees at ease so that they “feel free” to ask questions (supervisees appreciated that supervisors spoke “like a friend”); and being diplomatic and communicating about problems gently. For example, a supervisee and laboratory technician in Zambia noted: “Here you are lagging behind. They’re able to explain it in a nice way.” (FGD 13-01)

Supervisors’ attitudes were appreciated by supervised clinicians and mentioned frequently as differentiating the OTSS+ approach from other supervisors or supervision approaches in their countries. These attitudes included 1) humility (the supervisor is “democratic,” not a “know-it-all,” and appreciates he or she can also learn from the supervisee), 2) helpfulness (the supervisor comes with an attitude of improvement and doesn’t aim to “find fault,” but rather to mentor, “show how-to,” discuss, share knowledge, and coach supervisees to identify and solve problems); and 3) being positive, encouraging, and supportive (the supervisor motivates and reassures health facility staff, also shares positive feedback and avoids causing frustration).

*Before, as soon as they came, they threatened you, so you forgot what you knew. Now, they tell you, “Go ahead. You can also teach me. I am learning like you.” So we feel at ease. We are liberated. (Male, Nurse and Supervisee, Cameroon [FGD 20-05])*

*Even if what he says isn’t right, you don’t have to put him down and say it’s wrong. You accept and then you make corrections. And so, when you work in this dynamic, you retain what is positive. That’s the participatory approach. (Male, Government Stakeholder and Laboratory Supervisor, Cote D’Ivoire [KII 099])*

##### Length and maintenance of supervisory contact.

Supervisors and supervisees also underscored the importance of supervisors’ availability and ability “to take their time” or take the time that is “necessary.” Comments reflected both the overall time allotted for the visit—which ranged from half a day to 1 or 2 days, depending on a range of contextual factors, including health facility size, area of supervision, and supervision team size and composition—and supervisors’ effective use of that time to “go in depth” and exchange with health workers: “You can ask all the questions that you have because they are available.” Some supervisees also mentioned that they appreciated that supervisors made themselves available after the visit, providing their phone number or WhatsApp connection: “If there is a problem, you call him. He’ll talk to you and tell you what to do.” (Male, Nurse and Supervisee, Cameroon [FGD 20-05])

*There is already an element that is quite important. It is, first of all, the regularity of this supervision . . . . An agent who is supervised, who receives feedback, feels valued. And this can perhaps contribute significantly to his work. That is already quite important to me. (Male, Advisor and Other Stakeholder, Niger [KII 005])*

#### Joint problem solving and identification.

OTSS+ supervisors’ collaborative, problem-solving approach was felt to create an overall environment of openness, mutual learning, and trust. This collaborative approach was concretized via an action planning process that was seen as assigning responsibility and encouraging ownership of the agreed actions. Participants identified three factors that supported effective action planning. First, that the plan was drawn up jointly, in agreement with—and not imposed on—health facility staff. Gaps identified during the OTSS+ visit were typically discussed in a debrief session with the supervisees, in-charge, and supervisors, who would identify areas for improvement and agree on solutions jointly. Some participants noted that, optimally, the supervisees themselves would specify the solutions and time frame, helping to foster accountability for completion of actions. In addition, participants emphasized that supervisors or district/health area malaria focal points were, for their part, also responsible for following up and coaching or mentoring as required. Second, the action planning process was perceived to be effective because the health facility in-charge was present, which ensured the challenges and context within the health facility were understood. In some countries, district health management staff were involved systematically in OTSS+ visits. This was also perceived as encouraging ownership, supporting planning and implementation of malaria interventions, and facilitating follow-up of OTSS+ visits. Third, both supervisors and health facility participants perceived that action planning was effective because the actions or recommendations were “doable.” This was seen in terms of aligning with the available health facility workforce and avoiding assigning a task beyond the capacity of the health facility or the individual provider.

*[The action plan] provides ownership and assigns responsibility. . . . They sign for those gaps. You sign and they sign. So, there is no argument on the following time you go there to say, were these things done? Then, if they are not done, they need to provide an explanation. So, it is an effective way to benchmark or to check progress. (Male, Doctor and District Supervisor, Zambia [KII 064])*

*With the action plans that are developed, normally, we try to look at things that the facility can do, things that are within their ability to do. . . . The follow-up, we normally leave the follow-up to the districts’ health directorate level. . . . [E]ven subsequent OTSS [visits] that are done, we review the old plans to see whether they were actually implemented and what the gaps are that are still pertaining. (Male, Medical Superintendent and District Supervisor, Ghana [KII 043])*

### Perceived opportunities for improvement.

Participants identified five main areas for strengthening the effectiveness of the approach: refining digitized checklists, ensuring a sufficient pool of supervisors, devising approaches to prioritize health facilities in need, disseminating and following up action plans, and reviewing data and their use (Table 5).

#### Checklists.

Improvements to the OTSS+ approach over time have included streamlining and standardization of checklists. This streamlining was appreciated by participants from Ghana and Zambia, countries with historical experience using the OTSS approach. However, across most countries, supervisors observed there were still some context-specific and generalized refinements to the digitized checklists that could be made to improve usefulness or efficiency. One issue identified was the validity of percentile scoring for measures that were binary, or only covered two observations. Other issues related to skip patterns and redundancies resulting from the level of health facility being supervised. In addition, participants highlighted that the tools might be adapted for follow-up visits or contacts to reduce length, track follow-up visits, and flag improvements achieved or not achieved.

#### Pool of supervisors.

Participants from Cameroon and Niger, in particular, reported challenges resulting from an insufficient number of trained and experienced supervisors, which was attributed to factors such as attrition over time because of staff movements, unavailability as a result of conflicting agendas, and, in some areas, insufficient numbers of qualified health workers. The inability to maintain a pool of OTSS+ supervisors was reported sometimes to limit health facility coverage and follow-up, and was perceived to be a barrier to sustainability. Several stakeholders recommended training more supervisors from the district level, suggesting that this would improve the availability of supervisors, reduce costs, and promote ownership and engagement at the district level.

*The recommendation for me is first to increase the number of regional supervisors. Even the [numbers of] district supervisors also need to be increased. Because it would now be necessary to bring the district supervisors closer to the health facilities, so a district supervisor doesn’t have to travel 200 km to go and supervise a health facility. It’s tiring. And then it’s not effective. If the district supervisors are close to the supervisees, if they can regularly carry out the activity in their districts [that would be better]. I would normally like each technician to supervise in his district. (Male, Laboratory Technician and Regional Supervisor, Cameroon [KII 92])*

#### Health facility prioritization for initial and follow-up visits.

The OTSS+ approach introduced performance criteria to aid in selecting health facilities for OTSS+ supervision visits. Participants from most countries reported using a combination of performance or needs-based criteria to determine where support was most needed (e.g., health facilities with poor malaria indicators, high attendance, a larger catchment population, new health workers) with the final selection influenced by resource and other practical constraints. In many countries, participants noted a gap between the intended and actual approaches for health facility selection and follow-up visits. Participants reported they were unable to reach all prioritized health facilities or they struggled to determine whether to prioritize follow-up visits to supervised health facilities, frequently highlighting the importance of not “abandoning” a health facility versus prioritizing resources to target “new” health facilities. In Ghana and Zambia, participants reported that follow-up visits were, at times, deprioritized. In practice, participants in many countries reported that the decision to follow up with a health facility was determined by health facility performance, evidence of action plan implementation, supervisor availability, and resource constraints, leading to variation in the duration and frequency of support within and across countries.

*You don’t give up on a [health facility] . . . when you don’t go anymore . . . , after a while, the [health facility] falls back to the lowest level. . . . [E]ven when the structure has reached a good level of performance, you have to check from time to time to see if it is maintaining itself, if it has not dropped the good practices. (Female, Government Stakeholder and Clinical Supervisor, Côte D’Ivoire [KII 097])*

*To make me revisit the facility is when I am still concerned on their adherence to the treatment guidelines. Maybe they are undertesting or overtreating or otherwise when they don’t have adequate stock when the data [have] issues. And when sometimes there’s an increased number of patients for malaria compared to the normal number, . . . and I’m suspecting maybe an upsurge and they need some support. So those are some of the things that can make me revisit [a] facility again. (Female, Malaria County Coordinator and Supervisor, Kenya [KII 127])*

The OTSS+ approach typically plans for three follow-up visits conducted on a quarterly basis after the first visit. Most supervisors and managers confirmed that the optimal frequency for OTSS+ visits was quarterly, because it allowed sufficient contact between health facility staff and supervisors, although several suggested that a range of frequencies may be appropriate according to health facility performance. Participants noted that the amount and frequency of follow-up also depended on the type of actions defined in the action plan and approach used for follow-up.

Across countries, participants reported trying different follow-up approaches to address specific health facility needs and to prioritize OTSS+ resource use. For example, after conducting a first OTSS+ malaria-focused visit, it might be followed by a visit through a planned integrated supervision visit, either with the same or a different supervisor. Integration with other health areas was seen as one approach for promoting efficiency and improving reach. However, integration was often seen as both a strength and a threat, with participants citing a need to balance integration with a deeper, malaria-specific assessment. Some Zambian supervisees also preferred that the same supervisor conduct all visits to maintain the relationship. Another approach was to determine the weight required for the follow-up. Actions that were easier to resolve could be followed up via a phone call, whereas other actions might require more time and resources.

#### Action plan dissemination and follow-up.

Supervisors highlighted the importance of ensuring that both the health facility and the district have access to the action plan. Poor dissemination and availability of action plans at the health facility level was deemed to hamper implementation. Of the 12 health facilities visited by the study team, we found zero facilities with either a hard or electronic copy of the action plan available onsite. However, in FGDs in Cameroon, Ghana, and Niger supervisors and providers mentioned that action plans were sometimes copied in a designated notebook or that supervisors sent the plans by e-mail or WhatsApp after the visit. However, there was little mention of these plans being disseminated internally or made visible by the health facility team. Newer versions of the digital tool include an action planning module that allows for electronic documentation and dissemination of the plan at the time of the visit. However, participants reported that the action plans remained stored in the digital tool, to which the health facilities did not have access.

Weak action plan implementation was also perceived to limit effectiveness and threaten long-term engagement by stalling performance progress, wasting resources, and leading to a breakdown in the supervisor–supervisee relationship. Supervisors and managers reported two main challenges to action plan follow-up. The first was the absence of a system for tracking action status and unclear responsibilities of the different actors (supervisor, district, health facility) regarding verification of action completion. The second challenge related to insufficient or irregular follow-up visits as a result of resource constraints or supervisor availability (as described earlier regarding health facility prioritization), or a lack of supervisor continuity in areas where follow-up visits may be integrated or conducted by a different supervisor. More follow-up was perceived to increase the likelihood of adherence and completion of the action plan, and notable improvements in malaria service delivery between visits.

#### Data review and use.

Participants observed that OTSS+ data were used primarily by supervisors or district health managers to identify gaps in supplies and performance, provide on-the-spot capacity building, and follow-up on action plans for the health facility and district. Monthly or quarterly data review meetings at district or regional levels were described as the main forums to discuss OTSS+ visit results. A few participants noted there was an opportunity to increase the use of data generated by the OTSS+ approach at different levels in the health system to support strategic planning and allocation of resources.

## DISCUSSION

In this qualitative evaluation, the OTSS+ approach was widely perceived to be an effective approach for improving health facility and health worker performance in malaria service delivery and for improving indirectly data and supply management in a range of country contexts. These qualitative findings complement the quantitative evaluation of the OTSS+ visit data collected in Cameroon, Ghana, Niger, and Zambia, which found an association between successive OTSS+ visits to a health facility and improved health worker performance as assessed by the OTSS+ checklists.^22^ In our study, quality improvements were reported primarily with regard to effective service delivery, and were related to improved awareness, knowledge, and adherence to case management guidelines and diagnostic protocols. In addition, health workers reported increased confidence and motivation after OTSS+ visits, which are important outcomes in contexts confronted with high rates of health worker attrition. Other studies^1^^,^^15^ have found that formal supportive supervision is linked to increased health worker job satisfaction, motivation, or likelihood to remain in one’s post. Madede et al.^24^ found that supervision helped cultivate a more open and inclusive work environment, and that health workers felt there was space “to express their ideas and be heard.” In our study, provider satisfaction and motivation were derived in part from a sense of providing quality care and seeing satisfied patients. Importantly, providers also perceived improvements in the quality of patient–provider interactions, suggesting the OTSS+ approach encouraged the adoption of more people-centered care practices—a key element of quality health care.^25^

The OTSS+ approach was perceived to be a “holistic” process of improvement, suggesting that both supervisors and supervisees integrated the notion of a continuous process of improvement. Perceived effectiveness was driven primarily by the relevance and appropriate adaptation of checklists, the quality of supervisor–supervisee interactions, and a sense of joint responsibility generated through a collaborative approach and action planning. These findings are consistent in part with the limited evidence on what constitutes effective supportive supervision.

The use of standardized checklists has been shown to be effective for guiding systematic observation to measure performance^26^^,^^27^ and may be useful for facilitating data capture and use. However, a systematic review of 81 studies from 36 countries by Rowe et al.^9^ found that use of a checklist in itself during supervision visits was not associated with effectiveness. Participants in our study felt the checklists were relevant, and they were able to adopt and adapt the checklists in line with the needs of their context, reporting a sense of ownership for the process.

A recent review^28^ of successful supervision approaches found that they relied frequently on digital tools, which were integrated into existing data systems and informed by health system data. In our study, participants valued the use of digital tools, which was perceived to enhance objectivity and transparency, but reported that the use of OTSS+ data at subnational health system levels for malaria programming could be strengthened.

Studies have emphasized the overall importance of the quality of supervisor–supervisee interactions,^2^ and the importance and positive experience of onsite mentoring and coaching visits to individual health facilities.^29^ Participants in our study appreciated the onsite provision of coaching, mentoring, and other support provided by qualified and skilled supervisors who were focused on problem solving and who had enough time to “go in depth.” Although countries used different types of support (coaching, mentoring, training), we found no strong themes with regard to valuing a particular method or type of support, as long as it was in person, onsite, and appropriate to the context.

Many of the qualities and capacities ascribed to supervisors in our study reflected those that have been defined variously as making supervision “supportive.” Supervisors and supervisees both articulated supervisor attitudes and behaviors using a vocabulary that suggested deep knowledge and experience with engaging in respectful, two-way exchange focused on improvement, in stark contrast to approaches focused on inspecting, finding wrongdoing, and controlling.^13^ These attitudes and methods were perceived to differentiate the OTSS+ approach from other supervision approaches, both historical and contemporary, suggesting effective selection and training of OTSS+ supervisors, or suggesting that attitudes within health systems may have evolved more broadly in some contexts.

In our study, participants did not relate OTSS+ effectiveness strongly with the number of visits or visit frequency. They did perceive both the interaction length and follow-up (both intended and realized) to be a component of the quality of the interaction. Although we found participants across countries shared a common theoretical notion of the optimal frequency (i.e., quarterly), in most countries this intended schedule was difficult to operationalize as a result of planning and resource constraints. Some countries simply deprioritized follow-up visits in favor of reaching “new” health facilities; few participants expressed satisfaction that they were able to implement their health facility selection and follow-up approaches as desired. The literature on defining the right amount of support is similarly unclear. Desta et al.^30^ found that the optimal duration and frequency of supportive supervision visits to reach and maintain competency levels in facility-based primary health-care services in Ethiopia was five visits, separated by 6 to 9 months. In a systematic review of studies, Rowe et al.^9^ found the effects of supervision frequency (the number of visits per year) and dose (the number of supervision visits during a study) were unclear. It may be that the optimal frequency and duration of support is context specific and will vary in different places. Quantitative evaluation of OTSS+ data suggested that the duration required to reach competency varies by domain, even possibly within a health facility.^22^ Other studies have noted the relative importance of the quality of interactions over the frequency of visits. At the community level, a review by Hill et al.^31^ found that improving supervision quality has a greater impact than increasing frequency of supervision alone. The limited long-term evaluation of these kinds of approaches also makes it difficult to determine whether gains are sustained over time, with or without follow-up visits.

Although the provision of onsite support was highly valued, we found some evidence to suggest that it may be the follow-up contact itself (any type of contact) that may be critical for catalyzing improvements. Awareness that someone is following up and checking in appeared to promote accountability for and maintain attention on agreed actions. However, our evaluation of follow-up was limited by the absence of agreed definitions on what constitutes follow-up, systematic tracking of all follow-up contacts, and electronic recording and visualization of follow-up results. Further review and investigation of the different types of follow-up mechanisms (i.e., in-person return visits conducted by the same supervisor, integrated visits conducted by a different supervisor, or phone based) might help to clarify the relative value of the type, duration, and frequency of supervisory contacts.

In their recent systematic review, Rowe et al.^9^ found that having supervisors engage in problem solving with health-care providers was associated with greater mean effectiveness of supportive supervision. We found that joint review, problem solving, and action planning were highly valued, but that dissemination and follow-up of action plans remain persistent problems. An evaluation of OTSS supervisors at the end of the MalariaCare Project found similar perceptions that follow-up on action plans was inconsistent,^17^ and a recent study^32^ of supportive supervision in South Sudan found that action plans were followed up inadequately as a result of insufficient funding.

Avortri et al.^2^ called for a stronger focus on onsite, health facility–led (internal) supportive supervision, suggesting that a shift to building capacity at the lower levels of service delivery would help reduce the systemic and logistical challenges that hinder implementation. Although we found variations in implementation by country and even within countries, the OTSS+ approach is typically initiated at the regional level of the health system and, in practice, countries used a mix of approaches to identify health facilities for OTSS+ visits. Study participants emphasized the importance of involving district health management teams or their equivalent in OTSS+ visits and recommended training more supervisors at the district level. Other evidence has suggested that peer-to-peer learning is highly valued^3^; however, we found little mention of peer-to-peer exchange with the OTSS+ approach. When possible, shifting quality improvement processes to the district and health facility levels might encourage greater ownership; more self-assessment, peer-to-peer learning, and internal supervision; and perhaps make more efficient use of human and financial resources, allowing for wider or a more sustained reach of health facilities in need of support. More internally driven quality improvement might also incorporate systematic community outreach and engagement in quality improvement processes. The OTSS+ approach was designed originally to target support to the health facility level. However, some countries, such as Cameroon, have begun expanding the OTSS+ approach to the community level, where a large proportion of fever cases are first seen by community health workers. Building health facility–level capacity to support community health workers more effectively is likely crucial for improving the overall quality of malaria services and patient experience across the care continuum.

These factors suggest the overall importance of context-specific adaptations, not only of the OTSS+ checklists and tools, but also for OTSS+ visit planning and follow-up processes.

Our study had a few limitations. First, the retrospective design across diverse contexts limited our ability to assess comparatively the contribution of complementary approaches within individual countries, variation in implementing partners, or other important context-specific characteristics. We used probing techniques to clarify the influence of other factors that may have contributed to improvements. Second, data were collected from a small sample of OTSS+ health facilities in the four targeted countries. This was due to the need to balance priority areas of inquiry with operational constraints, and to the small number of health facilities that had undergone multiple OTSS+ visits in some countries. Smaller sampling frames in Ghana and Zambia (resulting from the smaller number of health facilities receiving multiple visits) mean these samples may be less representative of the full range of health facilities in those countries and may overrepresent those health facilities that did not demonstrate substantial improvement or were perceived to need significant support after the initial OTSS+ visit. Third, respondents may have been inclined to share predominantly positive viewpoints, particularly if they felt they had a stake in the evaluation findings. We attempted to minimize bias through clear statements on the study intent, scope, and use of findings; a combination of onsite and offsite data collection; and triangulation of perspectives to confirm and enrich our interpretation of themes emerging from the data. Last, patient and caregiver perspectives were not included in our evaluation. Exploration of their views regarding quality-of-care improvements, such as changes in provider behavior and quality of patient–provider interactions, health facility reception, patient flow, organization of care, time spent with the provider, information about their care, opportunities to raise concerns, and explanation of treatment after OTSS+ visits would be important for confirming that quality improvements were experienced by care seekers, in line with the principles of patient-centered care.^25^

## CONCLUSION

The OTSS+ approach was perceived to be a useful quality improvement approach for malaria services in a range of country contexts. Findings confirm the importance of the quality of supervisory contact and on-the-spot problem solving. Planning and health facility targeting require further context-specific attention to ensure the right health facilities are being reached and adequately followed up for long-term effect. To that end, further investigation to understand the optimal duration, frequency, and type of follow-up supervisory contacts in different contexts may be helpful. In addition, continued attention to embedding quality improvement processes at lower levels of the health system, and improved dissemination and follow-up of action plans may support increased effectiveness and efficiency. Future evaluations of similar quality improvement interventions should incorporate patient perspectives, in line with the principles of patient-centered care.
